# Roles of G-protein coupled receptors and mechanosensitive ion channels in pressure-induced chronotropy of lymphatic vessels

**DOI:** 10.1101/2025.10.07.681001

**Published:** 2025-10-07

**Authors:** Michael J. Davis, Hae Jin Kim, Min Li, Jorge A. Castorena-Gonzalez, Soumiya Pal, Timothy L. Domeier, Joshua P. Scallan, Scott Earley, Scott D. Zawieja

**Affiliations:** 1 Dept. of Medical Pharmacology & Physiology, University of Missouri, Columbia MO; 2 Dept. of Pharmacology, Tulane University School of Medicine, New Orleans LA; 3 Dept. of Molecular Pharmacology & Physiology, University of South Florida, Morisani School of Medicine, Tampa FL; 4 Department of Pharmacology and Physiology, University of Rochester Medical Center, Rochester, NY

**Keywords:** TRP channels, PIEZO1, ANO1, G proteins, mechanotransduction

## Abstract

Active lymph pumping relies on the spontaneous contractions of collecting lymphatic vessels, with frequencies that are exquisitely sensitive to changes in intraluminal pressure. This homeostatic and mechanosensitive mechanism, termed pressure-induced lymphatic chronotropy, enables lymph transport to be matched to the filling state of the lymphatic capillaries. We investigated the mechanistic basis of pressure-induced chronotropy using ex vivo contraction assays of mouse popliteal collecting vessels, in which contraction frequency increased >10-fold with a 5 cmH_2_O pressure change. The contractile, electrophysiological and transcriptional similarities between lymphatic muscle and arterial smooth muscle led us to hypothesize that pressure-dependent chronotropy shares a parallel signaling process to pressure-induce arterial depolarization/constriction. Thus, we investigated two major mechanisms: 1) pressure-induced activation of mechanosensitive cation channels, including TRPC6, TRPM4, PKD1/2, TRPV2 and ENaC, and 2) mechano-activation of GNAQ/GNA11-coupled GPCRs that would generate second messengers to activate those channels. We combined contraction assays with scRNAseq analysis of the respective targets and made maximum use of transgenic mice to avoid non-specific effects of pharmacological inhibitors, particularly those used to block TRP channels. Our findings rule out significant roles for TRP and other mechanosensitive channels implicated in myogenic constriction, as well as channels implicated in ionic pacemaking of other tissues, and instead support a scheme whereby mechano-activation of GNAQ/GNA11-coupled GPCRs generates IP_3_, which induces SR Ca^2+^ release through IP_3_R1 and drives depolarization through the activation of ANO1 Cl^−^ channels.

## Introduction

The lymphatic system is critically important for the maintenance of interstitial fluid balance. Because the Starling forces governing fluid movement across blood capillaries favor net filtration in most tissues (Levick & Michel, 2010), filtered fluid and protein will accumulate in the interstitium unless it is removed by the lymphatic system. Lymphatic capillaries are optimized to reabsorb that fluid, which can exceed 8 L·day^−1^ (Renkin, 1986). Once reabsorbed, lymph is transported by a combination of passive and active transport mechanisms through the collecting lymphatic vessel network, first to lymph nodes and eventually back into the venous system. Active lymph pumping relies on the spontaneous contractions of collecting lymphatic vessels—events that are initiated by action potentials (APs) in lymphatic muscle cells (LMCs). These contractions are relatively large in amplitude, with ejection fractions often exceeding 80% and sufficient to propel lymph through a system of one-way valves that ensure its unidirectional movement (Davis et al., 2025). Active pumping accounts for the majority of lymphatic transport in the lower legs of humans during quiet standing (Olszewski & Engeset, 1980) and facilitates lymph return against adverse hydrostatic pressure gradients that develop under gravitational loads (Davis et al., 2012; Scallan et al., 2016; Li et al., 2022). An important feature of the active lymph pump system is the exquisite sensitivity of the spontaneous contraction frequency of collecting lymphatic vessels to changes in their intraluminal pressure, enabling lymph transport to be matched to the filling state of the lymphatic capillary network (Scallan et al., 2012). This homeostatic mechanism is termed “pressure-induced lymphatic chronotropy” (Zawieja et al., 2019). In popliteal collecting vessels of the mouse hindlimb, the spontaneous contraction frequency can increase from 2 to 20 contractions·min^−1^ as pressure is elevated from 0.5 to 10 cmH_2_O, i.e., a 10-fold increase over the physiological pressure range of those vessels (Davis et al., 2020; Davis et al., 2023a; Davis et al., 2023b). Contraction amplitude increases modestly over a portion of the same pressure range (Scallan et al., 2012), but modulation of contraction frequency is the primary determinant of active lymph transport.

Pressure-induced chronotropy reflects an underlying mechanotransduction process that ultimately involves control of an intrinsic pacemaker in LMCs by intraluminal pressure. Although the specific force sensing mechanism is unknown, contractile, electrophysiological and transcriptional similarities between LMCs and vascular smooth muscle cells (VSMCs) support the hypothesis that pressure-dependent chronotropy shares a parallel signaling process to the myogenic response of VSMCs. The myogenic response refers to in rapid increase in arterial pressure induces distention of a small artery / arteriole, which then constricts over time until a new, stable diameter lower than the original diameter is reached (Davis, 2012). A similar constriction often occurs in collecting lymphatic vessels simultaneously with pressure-induced chronotropy (Davis et al., 2009). Pressure-induced arterial constriction is preceded by VSM depolarization (Harder, 1984) and several lines of evidence support a critical role for the activation of (putative) mechanosensitive cation channels in that process—in particular the involvement of three TRP channel family members: TRPC6, TRPM4 and PKD1/2.

The first evidence for a molecularly identified ion channel involved in myogenic constriction came from the demonstration that single channel currents in VSMCs isolated from rat cerebral arteries were activated by patch pipette suction and those currents, along with pressure-induced cerebral artery constrictions and depolarizations, were attenuated after downregulation of TRPC6with antisense oligonucleotides (Welsh et al., 2002). TRPM4 channels were likewise implicated after inward, TRPM4-like currents in rat pial artery myocytes were found to be activated by membrane stretch, and because antisense- or siRNA-mediated knock down of TRPM4 significantly attenuated pressure-induced depolarization and myogenic tone development in those arteries (Earley et al., 2004b; Gonzales et al., 2014). However, results from *Trpc6*^−/−^ and *Trpm4*^−/−^ mice tended to contradict the above studies in finding no differences or even *enhanced* arterial myogenic constriction in the global knock out animals (Dietrich et al., 2005; Mathar et al., 2010; Schleifenbaum et al., 2014). Evidence also supports roles for PKD2 (previously described as TRPP1) channels in myogenic constriction, but it, too, is controversial. Jaggar and colleagues reported that shRNA-mediated down-regulation of *Pkd2* attenuated pressure-induced depolarization and myogenic tone in rat pial arteries (Narayanan et al., 2013) and that pressure-induced constriction was blunted in hindlimb arteries isolated from Myh11-CreER^T2^;*Pkd2*^*f/f*^ (smooth muscle knockout, smKO) mice (Bulley et al., 2018). However, a study by Honoré and coworkers suggested that stretch-activated cation currents and myogenic constriction in mouse mesenteric artery smooth muscle were mediated by PKD1 channels (previously termed TRPP2), and that PKD2 channels played a counteracting role (Sharif-Naeini et al., 2009). Myogenic responses could not be tested in global *Pkd2* KO mice because constitutive deletion of *Pkd2* is embryonically lethal (Wu et al., 1998).

More recent work points to G-protein-coupled receptors (GPCRs) rather than TRP or other ion channels as the primary mechanosensing elements in the arterial myogenic response. Studies of cardiomyocyctes first demonstrated that the angiotensin receptor, AT_1_R, a GNAQ/GNA11-coupled GPCR, could be activated by mechanical stress through a mechanism independent of its ligand (Zou et al., 2004). Gudermann and coworkers provided compelling support for this mechanism using patch-clamped HEK293 cells, co-expressing AT_1_R and TRPC6 channels, with hypoosmotic swelling as a mechanical stimulus (Mederos y Schnitzler et al., 2008). Two critical findings were 1) that TRPC6 current was activated by hypoosmotic swelling *only* if the cells co-expressed *Agtr1* (Mederos y Schnitzler et al., 2008) and 2) that the swelling-induced currents were largely prevented by inverse AT_1_R agonists. *Agtr1* could be replaced by another GNAQ/GNA11-coupled GPCR, or *Trpc6* by another DAG-sensitive TRP channel (Mederos y Schnitzler et al., 2008). Subsequent studies from multiple laboratories, using selective pharmacological inhibitors of AT_1_R, acute *Agtr1* knockdown, and/or mice deficient in *Agtr1* or other GNAQ/GNA11-coupled GPCRs, confirmed that basic principle in VSMCs: that pressure-induced constriction of many different types of arteries is transduced primarily, or exclusively, by GNAQ/GNA11-coupled GPCRs (Blodow et al., 2014; Harraz et al., 2014; Li et al., 2014b; Schleifenbaum et al., 2014; Pires et al., 2017; Bjorling et al., 2018). Helix 8 in the GPCR c-terminus was subsequently identified as the essential structural motif endowing mechanosensitivity (Erdogmus et al., 2019). The collective data support a model in which phospholipase C is activated downstream from GNAQ/GNA11-coupled GPCRs in response to elevated pressure, leading to increased production of DAG and IP_3_/Ca^2+^ release, which activate TRPC6 and TRPM4 channels, respectively, to depolarize the cell and increase the open probability of L-type voltage-gated Ca^2+^ channels, thereby promoting global Ca^2+^ influx and contraction (Davis et al., 2023c). In a similar manner, TRPM4 and PKD2/2 channels might be activated by Ca^2+^ influx/release downstream from mechano-activation of a GPCR or another Ca^2+^-permeable ion channel such as PIEZO1 (Peyronnet et al., 2013) or TRPV4 (Swain & Liddle, 2021).

Whether a similar scheme is operative in LMCs is unknown. Each lymphatic contraction is preceded by a near-linear depolarization from the “resting” potential to threshold potential, termed the diastolic depolarization, which is the primary determinant of AP/contraction frequency (Zawieja et al., 2025). Thus, lymphatic pressure elevation manifests not as a stepwise increase in baseline potential LMCs (von der Weid et al., 2014; Davis & Zawieja, 2018) but rather as an increase in the diastolic depolarization slope and a reduction in time required to reach threshold for AP firing (Zawieja et al., 2018b; Zawieja et al., 2019). Previously, we found that regulation of the diastolic depolarization slope was mediated largely by activation of the Ca^2+^ activated chloride channel ANOCTAMIN 1 (ANO1, also referred to as TMEM16A). ANO1 is not intrinsically mechanosensitive, but is regulated by Ca^2+^ and, in LMCs specifically, by IP_3_ receptor 1-mediated Ca^2+^ release from sarcoplasmic reticulum (Zawieja et al., 2019; Zawieja et al., 2023), possibly downstream from one or more mechanosensitive GPCRs. However, other mechanisms are also involved because *Ano1* deletion from smooth muscle or pharmacological inhibition of ANO1 does not completely eliminate spontaneous APs or contractions (Zawieja et al., 2019). The primary goal of the present study was to test the roles of putative VSMC mechanosensitive ion channels, channels implicated in the regulation of pacemaking in other cell types, and GNAQ/GNA11-coupled GPCRs in pressure-induced chronotropy of popliteal lymphatic vessels. An advantage in studying this particular aspect of lymphatic vessel mechanotransduction is the high sensitivity of the spontaneous contraction frequency to pressure, increasing ~10-fold over only a 5 cmH_2_O pressure range in popliteal lymphatics compared to relatively modest levels of myogenic constriction (50% change) over a 60 mmHg pressure range, even in the most myogenically reactive arteries (Davis et al., 2023c). As part of our strategy, we sought to make maximum use of transgenic mice to avoid non-specific effects of pharmacological inhibitors, particularly those used to block TRP channels.

## Materials and Methods

### Protocol approval.

All procedures were reviewed and approved by the animal care committee at the University of Missouri and complied with the standards stated in the “Guide for the Care and Use of Laboratory Animals” (National Institutes of Health, revised 2011).

### Mice.

*WT* mice [C57Bl/6 (#000664) and SVF129 (#101043) strains], *Slc8a1*^−/−^ (#025943) mice and *AT*_*1a*_*R*^−/−^ (#002682) mice were purchased from The Jackson Laboratory (Bar Harbor, Maine). Myh11-CreER^T2^mice were gifts from Stefan Offermans (Max Plank Institute, GDR). Sperm from *Gna*_*12*_^−/−^*;Gna*_*13*_^*f/f*^ and *Gna*_*11*_^−/−^*;Gna*_*q*_^*f/f*^ mice were gifts from Stefan Offermanns, after which the respective mice were rederived at MMRC, Columbia, MO to generate Myh11-CreER^T2^*;Gna*_*13*_^*f/f*^*;Gna*_*12*_^−/−^ mice *and* Myh11-CreER^T2^*;Gna*_*q*_^*f/f*^*;Gna*_*11*_^−/−^ mice. *Trpc6*^−/−^ and *Trpc3*^−/−^ mice were gifts from Lutz Birnbaumer (NIH); *Trpc6*^−/−^*;Trpc3*^−/−^ double KO mice were generated by crossing those two strains. Myh11-CreER^T2^*;Kcnq4*^*f/f*^ *(encoding Kv7.4)* mice were a gift of Thomas Jentsch (Free Univ. of Berlin, GDR). *Piezo1*^*f/f*^ mice were a gift from Ardem Pataputian (UCSD) and were bred to Myh11-CreER^T2^ mice to generate Myh11-CreER^T2^*; Piezo1*^*f/f*^ mice. *Ano1*^*f/f*^ mice, generated in the lab of Jonathan Jaggar (Leo et al., 2021) were bred to Myh11-CreER^T2^ mice to obtain Myh11-CreER^T2^*;Ano1*^*f/f*^ mice. *Itpr1*^*f/f*^ mice were obtained from Ju Chen (UCSD) and bred to Myh11-CreER^T2^mice to generate Myh11-CreER^T2^*;Itpr1*^*f/f*^ mice. *Pkd2*^*f/f*^ mice were generated in the University of Maryland PKD Core by Jonathan Jaggar, as previously described (Bulley et al., 2018), and bred in the Jaggar laboratory to Myh11-CreER^T2^mice to generate Myh11-CreER^T2^*;Pkd2*^*f/f*^ mice. *Trpm4*^*f/f*^ mice were generated in the laboratory of Scott Earley and bred to Myh11-CreER^T2^ mice to produce Myh11-CreER^T2^*;Trpm4*^*f/f*^ mice. *Trpv4*^*f/f*^ mice were generated in the laboratory of Timothy Domeier (University of Missouri) and bred to Myh11-CreER^T2^ mice to produce Myh11-CreER^T2^*;Trpv4*^*f/f*^ mice. *Rosa26mTmG*^f/f^ mice were purchased from JAX (#007676) and bred to Myh11-CreER^T2^ and Myh11-CreER^T2^*;Ano1*^*f/f*^ mice to produce Myh11-CreER^T2^*;Rosa26mTmG*^*f/f*^ *and* Myh11-CreER^T2^*;Ano1*^*f/f*^*; Rosa26mTmG*^*f/f*^ mice.

For genotyping, genomic DNA was extracted from tail clips using the HotSHOT method. Genotypes were determined by PCR with 2x PCR Super Master Polymerase Mix (Catalog # B46019, Bimake, Houston, TX) according to the provider’s instructions. Mice containing Myh11-CreER^T2^ were induced with five consecutive daily injections of 100 mg tamoxifen (10mg/kg i.p.; in safflower oil). Those mice were studied a minimum of 2 weeks after induction. All floxed control mice were subjected to the same induction protocol. Because the Myh11-CreER^T2^ used for SM-specific deletion of floxed genes is carried on the y chromosome, only male mice were used for experiments involving Myh11-CreER^T2^ mice or their floxed controls. For other strains, both male and female animals were used.

### Vessel isolation, pressure myography, and data acquisition.

Mice were anesthetized with ketamine/xylazine (100/10 mg/kg, i.p.) and placed face down on a heated tissue dissection/isolation pad. The saphenous vein was exposed by a proximal-to-distal incision along the skin of the calf and the popliteal afferent lymphatic vessels on each side of the vein were isolated as previously described (Scallan & Davis, 2013). Each vessel was then pinned with short segments of 40 μm stainless steel wire onto the SYLGARD-coated surface of a dissection chamber filled with BSA-supplemented Krebs buffer at room temperature. Once secured, the surrounding adipose and connective tissue were removed by microdissection. An isolated popliteal lymphatic vessel was then transferred to a 3-mL observation chamber, cannulated, pressurized to 3 cmH_2_O using two glass micropipettes (50–60 μm outside diameter) and moved to the stage of a Zeiss inverted microscope. Lymphatic segments used in these studies typically contained a single valve. Polyethylene tubing attached to the back of each glass micropipette was connected to a two-channel computerized pressure controller (Scallan et al., 2013). To minimize diameter-tracking artifacts associated with longitudinal bowing at higher intraluminal pressures, input and output pressures were briefly set to 10 cmH_2_O at the beginning of every experiment, and the vessel segment was stretched axially to remove longitudinal slack. The lymphatic vessel was then allowed to equilibrate at 37°C with inflow and outflow pressures set to 3 cmH_2_O. Constant exchange of Krebs buffer was maintained using a peristaltic pump at a rate of 0.5 mL/min. After temperature stabilized, popliteal lymphatics typically began to exhibit spontaneous contractions within 10–15 min and stabilized in a consistent contraction pattern within ~30 minutes. Custom LabVIEW programs (National Instruments; Austin, TX) acquired real-time analog data and digital video through an A-D interface (USB-6216, National Instruments) and detected the inner diameter of the vessel at 30 fps using a Basler A641fm firewire camera (Davis, 2005). Videos of the contractile activity of lymphatic vessels were recorded for further analysis, if needed.

### Assessment of ex vivo contractile function.

The contractile parameters of each vessel were characterized at different levels of intraluminal pressure spanning the physiological range from 0.5 to 10 cmH_2_O (in successive steps as follows: 3, 2, 1, 0.5, 3, 5, 8, and 10 cmH_2_O). Spontaneous contractions were recorded at each pressure in typical intervals of 2 minutes, although a period of 4–5 min was sometimes required at the lowest pressure to obtain multiple contractions. During the pressure response protocol, both the input and output pressures were maintained at equal levels so that there was no imposed pressure gradient for forward flow. At the end of every experiment, all vessels were equilibrated by perfusion with calcium-free Krebs buffer containing 3 mM EGTA for 30 minutes, after which passive diameters were obtained at each level of intraluminal pressure.

### Contractile function parameters.

Once an experiment was completed, internal diameter traces and/or 30-fps brightfield videos of spontaneous contractions were analyzed using custom-written LabVIEW programs (Davis, 2023b, a) to detect end diastolic diameter (EDD), end systolic diameter (ESD), and contraction frequency (FREQ, computed on a contraction-by-contraction basis), with each parameter averaged over a 2–5 min period. These data were used to calculate the following commonly reported parameters that characterize the contractile function of lymphatic vessels:

(1) Amplitude=EDD-ESD

(2) $$\text{Normalized}\;\text{Contraction}\;\text{Amplitude}=\left(\frac{\text{EDD}-\text{ESD}}{D_{\text{MAX}}}\right)\times 100$$

(3) $$\text{Ejection}\;\text{Fraction}\;(\text{EF})=\left[\frac{{EDD}^{2}-{ESD}^{2}}{{EDD}^{2}}\right]$$

(4) $$\text{Tone}=\left(\frac{D_{\text{MAX}}-EDD}{D_{\text{MAX}}}\right)\times 100$$

(5) FractionalPumpFlow(FPF)=EF·FREQ

where $D_{\text{MAX}}$ represents the maximum passive diameter (obtained after incubation with calcium-free Krebs solution) at a given level of intraluminal pressure. When frequency was zero, a value for amplitude was omitted. The relative paucity of some amplitude data at low pressures in some genotypes reflects the fact that this parameter could not be determined when frequency was zero. Each of the contractile parameters represented the average of all the recorded contractions during the measurement period at each intraluminal pressure.

### Analysis of pressure-induced chronotropy.

**Figure 1.**
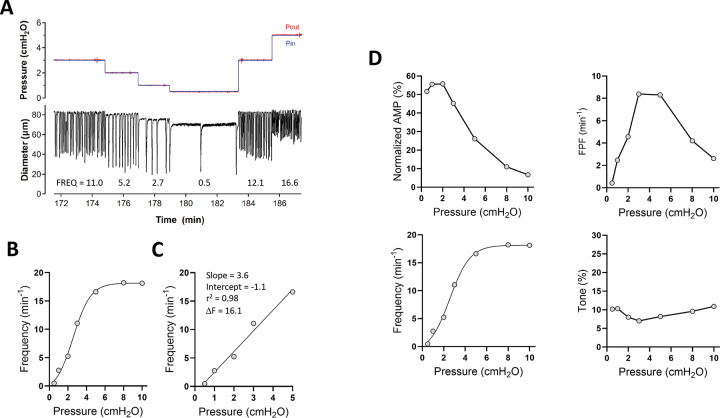
Quantification of the F-P relationship. Steps for quantitative assessment of pressure-induced chronotropy. **A**) Representative recording of pressure and diameter changes in a WT popliteal lymphatic vessel during inflow (Pin) and outflow (Pout) pressure steps between 0.5 and 5 cmH_2_O. Frequencies stated at the bottom of the trace were determined off-line using a custom peak detection program written in LabVIEW. **B**) Plot of frequency vs pressure for the vessel in A, showing how frequency reaches a plateau above 5 cmH_2_O. **C**) Fit of the frequency data with a first-order equation and calculation of the frequency difference (ΔF) between 5 and 0.5 cmH_2_O. **D**) Summary of contraction data for this representative WT vessel over the stated pressure range; see Methods for description of parameter calculations.

Quantification of the frequency-pressure (F-P) relationship for comparisons across genotypes required additional analysis steps, as illustrated in Fig. 1. Raw recordings of pressure and diameter (Fig. 1A) show the responses of a WT popliteal vessel to descending pressure steps from 5 to 0.5 cmH_2_O, with the average frequency stated for each pressure. The frequency data were then plotted as a function of pressure (Fig. 1B). As evident from the behavior of this representative vessel, frequency tended to plateau above 5 cmH_2_O, so the subset of data in which the response was nearly linear over the pressure range 0.5–5 cmH_2_O was fitted with a first-order polynomial to obtain the slope and intercept (Fig. 1C). As an alternative method, we considered calculating the ratio of the respective contraction frequencies at 0.5 and 5 cmH_2_O; however, in many cases no contractions occurred at P = 0.5 cmH_2_O, which would equate to a ratio of infinity. To avoid this problem, we computed the difference (ΔF) between the frequencies at 5 and 0.5 cmH_2_O. This approach gave consistent values that, like the slope, could detect blunting of the F-P relationship, but in essentially every case the results matched those of the curve fitting procedure; thus, only the F-P slope was used for statistical analyses and shown in subsequent figure plots. To enable a more comprehensive assessment of contractile function, full plots of amplitude, frequency, FPF and tone were also plotted as a function of pressure for each genotype (Fig. 1D).

### FACS.

Inguinal-axillary lymphatic vessels from tamoxifen-treated Myh11-CreER^T2^*;mTmG*^*f/f*^ *and* Myh11-CreER^T2^*;Ano1*^*f/f*^*;mTmG*^*f/f*^ mice were dissected and cleaned of fat and connective tissue as described previously (Zawieja et al., 2018a). Cleaned vessel segments were transferred to a 1-ml tube of low-Ca^2+^ PSS containing (in mM): 137 NaCl, 5.0 KCl, 0.1CaCl_2_, 1.0 MgCl_2_, 10 HEPES, 10 Glucose, and 1 mg/ml BSA at room temperature for 10 min. The solution was decanted and replaced with a similar solution containing 26 U/ml papain (Sigma, St. Louis, MO) and 1 mg/ml dithioerythritol. The vessels were incubated for 30 min at 37°C with occasional agitation, then transferred to a new tube containing low-Ca^2+^ PSS containing 25 mg/ml collagenase H (FALGPA U/ml, Sigma), 0.7 mg/ml collagenase F (Sigma), 20 mg/ml trypsin inhibitor (Sigma), 1mg/ml elastase (Worthington), and incubated for 6 min at 37°C. The dispersed cells were sedimented by centrifugation (300 g, 4 min), resuspended in 0.6 ml PSS containing 1mM Ca solution, and filtered through a 35-μm mesh nylon filter to obtain a single cell suspension. GFP+ LMCs were sorted by fluorescence-activated cell sorting (FACS) using a Beckman-Coulter MoFlo XDP instrument with excitation laser (488 nm) and emission filter (530 ± 40 nm), a 70 μm nozzle, a sheath pressure of 45 psi and sort rate of 100 events per second. Sorting was performed at the Cell and Immunobiology Core facility at the University of Missouri.

### RNA isolation and quantitative, real-time PCR.

Total RNA was extracted from FACS-sorted cells using the Arcturus PicoPure RNA isolation kit (ThermoFisher Scientific, Waltham, MA) with on-column DNase I treatment (Qiagen, Valencia, CA) according to manufacturer’s instructions. RNA was eluted with nuclease-free water. Purified RNA was transcribed into cDNA using High-Capacity cDNA Reverse Transcription kit (ThermoFisher Scientific, Waltham, MA). Real-time PCR (qPCR) was performed on cDNAs prepared from each sample using 2x PrimeTime Gene Expression Master Mix (IDT, Coralville, IA) with predesigned TaqMan probes as listed in Suppl. Table 1 (IDT, Coralville, IA). Real-time PCR protocols were as follows: preheating at 95°C for 3 min, 45 cycles of two-step cycling of denaturation at 95°C for 15 sec and annealing/extension steps of 30 sec at 60°C. Data collection was carried out using a Bio-Rad CFX 96 Real-Time Detection System (software version Bio-Rad CFX Manager 3.1; Bio-Rad, Hercules, CA, USA). For analysis, the result was expressed as a ratio of target gene/reference gene (β-actin).

### scRNAseq analysis.

A total of 10 *Rosa26mTmG* mice (5 males and 5 females), without Cre and without tamoxifen treatment were used for scRNAseq analysis. Inguinal-axillary lymphatic vessels (IALVs) from both sides of each mouse were isolated and cleaned of connective and adipose tissue. The pooled vessels were digested into a single cell suspension and the cells were kept on ice until all tissues had been processed. Cells from all vessels were combined and sorted for tdTomato expression to remove debris and concentrate the cells for downstream single cell 3’ RNA-Seq libraries creation with 10x Genomics Chromium Chip and Chromium Next GEM Single Cell 3’ RNA-Seq reagents (Zawieja et al., 2025). Samples were sequenced with the NovaSeq 6000 S4-PE100 flow cell.

Mus musculus genome GRCm39 and annotation GTF (v106) from Ensembl (https://useast.ensembl.org/Mus_musculus/Info/Index) were used to build the reference index and the reads were processed using Cell Ranger (v7.0.1) using the default parameters. The quality control and filtering steps were performed using R (v4.2.1; https://www.r-project.org/). Ambient RNA was removed from the Cell Ranger output with SoupX (Young & Behjati, 2020). A doublet score for each cell was estimated using scDBlFinder [v1.12.0; (Germain et al., 2021)]. Non-expressed genes (sum zero across all samples) and low-quality cells (>10% mitochondrial genes, < 500 genes, < 1,000 UMIs per cell and doublet score <0.5) were removed with custom R scripts. Cells passing filtering were normalized/scaled (SCTransformation), dimensionally reduced (t-distributed stochastic neighbor embedding (t-SNE), uniform manifold approximation and projection (UMAP)) clustered, and hierarchically analyzed with Seurat (Hao et al., 2021; Hao et al., 2024) using the default parameters. Marker gene expression profile on cell clusters and gene co-expression were visualized using Seurat and ShinyCell R app (Ouyang et al., 2021 PMID: 33774659). The raw scRNAseq dataset is available on the NIH GEO #GSE277843 (eLIFE ref).

### Solutions and chemicals.

Krebs buffer contained: 146.9 mM NaCl, 4.7 mM KCl, 2 mM CaCl_2_·2H_2_O, 1.2 mM MgSO_4_, 1.2 mM NaH_2_PO_4_·H_2_O, 3 mM NaHCO_3_, 1.5 mM Na-HEPES, and 5 mM D-glucose (pH = 7.4). An identical buffer was prepared with the addition of 0.5% bovine serum albumin (“Krebs-BSA”). During cannulation Krebs-BSA buffer was present both luminally and abluminally; however, during the experiment the abluminal solution was constantly exchanged with plain Krebs. Ca^2+^-free Krebs was Krebs with 3 mM EGTA replacing CaCl_2_·2H_2_O. All chemicals were obtained from Sigma-Aldrich (St. Louis, MO), with the following exceptions: BSA (US Biochemicals; Cleveland, OH), MgSO_4_ and Na-HEPES (ThermoFisher Scientific; Pittsburgh, PA), ivabradine, zatebradine and losartan (Tocris). Amiloride, benzamil, ivabradine, zatebradine, BaCl_2_ and losartan were dissolved in water to make stock solutions. Ani9, tranilast and SET2 were dissolved in DMSO to make stock solutions. Each inhibitor was then further diluted in Krebs solution to reach the final concentrations stated.

### Statistical analysis.

The data were analyzed in LabVIEW, compiled in Excel and statistical analyses were performed using Prism (v.10.2; Graphpad, San Diego, CA). Statistical differences in the slopes of the F-P relationships between two groups were assessed by an unpaired, two-tailed t-test, and differences between three or more groups were tested using a one-way ANOVA with Dunnett’s post-hoc tests. Comparisons between contraction parameters as a function of pressure were made using two-way or mixed-model ANOVAs with repeated measures and Sidák’s post-hoc tests. Data are plotted as mean ± SEM. Significance levels (compared to the control group) are either stated or indicated as follows: *p<0.05; **p<0.01; ***p<0.001; ****p<0.0001; “n.s.” p≥0.05, with the color-coding of the symbols matching the respective group. In the figure legends, N refers to the number of animals and *n* refers to the number of vessels per group.

## Results

### Role of ANO1 in pressure-induced chronotropy.

**Figure 2.**
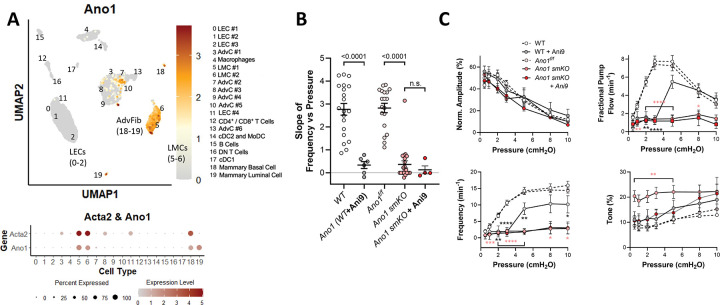
Consequences of ANO1 deletion and/or inhibition. **A**) UMAP plot of *Ano1* expression in the various cell populations within the walls of murine IALVs that were thoroughly cleaned prior to dissociation and scRNAseq analysis, as described in (Zawieja et al., 2025). The LMC cluster (composed of at least two subclusters of 978 cells) was identified by the expression of canonical smooth muscle cell markers *Acta2* (SM α-actin), *Myh11* (myosin heavy chain 11), *Itga8* (integrin alpha 8). Gray color in this and subsequent figures represents *Acta2* expression, red/brown in this panel indicates *Ano1* expression. Bubble plot shows Ano1 expression in the LMC and other cell clusters in terms of percent cells in an individual cluster expressing *Ano1* (size of dot) and the *Ano1* expression level relative to *Acta2* (color of dot). **B**) Slope of frequency vs pressure (F-P) for WT (C57Bl/6) vessels with / without treatment by the ANO1 inhibitor Ani9 (1 μM), for *Ano1*^*f/f*^ control vessels, for *Ano1 smKO* vessels and for the latter treated with Ani9. **C**) Summary plots of contraction parameters as a function of pressure for the 5 respective groups of vessels. WT N=15, n=20; WT+Ani9 N=5, n=6; *Ano1 smKO* N=14, n=23; *Ano1*^*f/f*^ N=8, n=16.

Single cell RNAseq analyses from dissected IALVs revealed 19 distinct cell clusters (Fig. 2A), segregating into prominent clusters/subclusters of lymphatic endothelial cells (LECs), LMCs, adventitial cells (AdvCs) and immune cells. Further analysis confirmed that *Ano1* was expressed at moderate levels (relative to *Acta2*) in >75% of LMCs and to a lesser extent in AdvCs, but not in LECs (Fig. 2A). AdvCs contained subclusters of adventitial fibroblasts and other cell types expressing multiple stem cell markers (Zawieja et al., 2019).

Previously, we reported that ANO1 inhibition or SM-specific *Ano1* deletion caused a nearly complete abrogation of pressure-induced chronotropy in mouse IALVs. However, the contraction frequency of IALVs changes only ~1.7 fold over the pressure range from 0.5 to 10 cmH_2_O (Zawieja et al., 2019), compared to the much larger dynamic range (≥ 10-fold) characteristic of popliteal lymphatics (Fig. 1). Thus, we used popliteal lymphatics throughout this study to assess pressure-induced chronotropy, in anticipation that we would be able to detect even subtle changes in the response after deletion of specific genes. The F-P analysis for 20 WT popliteal lymphatic vessels is shown in Fig. 2. C57Bl/6 mice served as the WT control group for many comparisons because all transgenic strains used in the present study (with one exception) were derived from, or backcrossed into, that background. The average slope (± SEM) of the F-P relationship between 0.5 and 5 cmH_2_O was 2.8 ± 0.3 for WT vessels (Fig. 2B), equating to an average frequency difference of 12.1 ± 1.0 contractions·min^−1^ over that pressure range. The average r^2^ value for the linear regressions was 0.93 ± 0.02, indicating a consistently high quality of the curve fits and confirming that the slope was approximately linear between 0.5 and 5 cmH_2_O. The role of ANO1 in pressure-induced chronotropy of mouse popliteal lymphatic vessels was then tested after pharmacologic inhibition of ANO1 with Ani9, one of the most selective small molecule inhibitors of ANO1 (Hwang et al., 2016). Initial assessment of the Ani9 concentration-response relationship (from 30 nM to 10 μM) in WT popliteal vessels revealed that 3 μM Ani9, the concentration previously used to inhibit ANO1 in IALVs (Zawieja et al., 2019; Zawieja et al., 2023), did indeed reduce the basal contraction frequency of popliteal lymphatics (in the lower pressure range), but also caused a loss of tone and marked reduction in their contraction amplitude (not shown)—suggestive of possible off-target effects; therefore, for subsequent F-P protocols we used a lower concentration (1 μM) of Ani9, which had insignificant effects on contraction amplitude and tone (Fig. 2C) but significantly reduced the average slope of the F-P relationship from 2.8 ± 0.3 to 0.3 ± 0.2 (p<0.001; Fig. 2B).

We then tested the effects of SM-specific *Ano1* deletion by comparing the F-P relationship of popliteal lymphatic vessels from tamoxifen-treated Myh11-CreER^T2^*;Ano1*^*f/f*^ (*Ano1 smKO*) mice to *Ano1*^*f/f*^ control mice. The results are also shown in Fig. 2B. The average slope of the F-P relationship between 0.5 and 5 cmH_2_O for *Ano1 smKO* vessels was 0.4 ± 0.2, significantly lower (p<0.001) than that (2.8 ± 0.2) for *Ano1*^*f/f*^ vessels. Complete sets of contraction data for untreated and Ani9-treated WT popliteal lymphatics, Myh11-CreER^T2^*;Ano1*^*f/f*^ and *Ano1*^*f/f*^ popliteal vessels, are shown in Fig. 2C. The frequency of Ani9-treated vessels was significantly reduced at most pressures but increased at higher pressures. Normalized contraction amplitudes were unchanged by Ani9. Tone was slightly but not significantly higher in Ani9-treated vessels. After *Ano1* deletion, frequency was reduced to <3 contractions·min^−1^ at all pressures, in contrast to Ani9-treated WT vessels in which frequency was comparably reduced at low pressures but not at elevated pressures (Fig. 2C). *Ano1 smKO* vessels had significantly elevated levels of tone at 5 of 7 pressures, suggesting that Ani9 treatment may have interfered with tone development in a way that *Ano1* deletion did not. Collectively, these results show that deletion / inhibition of ANO1 significantly impaired pressure-induced chronotropy in popliteal lymphatic vessels; however, SM-specific *Ano1* deletion failed to completely abolish spontaneous contractions in 16 of 21 *Ano1 smKO* vessels. To test the possibility that residual pacemaking activity in *Ano1 smKO* vessels might be due to incomplete recombination by the inducible Myh11-CreER^T2^ driver, *Ano1* mRNA levels in IALVs were quantified using qPCR analysis. Smooth muscle cells were digested from IALVs of Myh11-CreER^T2^*;mTmG*^*f/f*^ or Myh11-CreER^T2^*;mTmG*^*f/f*^*;Ano1*^*f/f*^ mice and purified by FACS using the GFP signal. *Ano1* message (referenced to α-actin levels) in LMCs from Myh11-CreER^T2^*;mTmG*^*f/f*^*;Ano1*^*f/f*^ vessels was undetectable compared to that in LMCs from Myh11-CreER^T2^*;mTmG*^*f/f*^ vessels (0 ± 0 and 1006 ± 80, respectively; mean ± SD, n=3). As a further test for possible residual ANO1 activity in *Ano1 smKO* vessels, we treated another group of *Ano1 smKO* vessels with Ani9 (1 μM), and those results are shown in Fig. 2B–C. Pacemaking activity persisted even after combined deletion and inhibition of ANO1, without significant further reductions in frequency at any pressure, suggesting that residual pacemaking in *Ano1* KO vessels was *not* due to incomplete ANO1 knock down. Collectively, these results point to ANO1 as the major component of pressure-induced lymphatic chronotropy but also to the involvement of one or more additional mechanisms, possibly involving other LMC ion channels.

### Role of PIEZO1.

**Figure 3.**
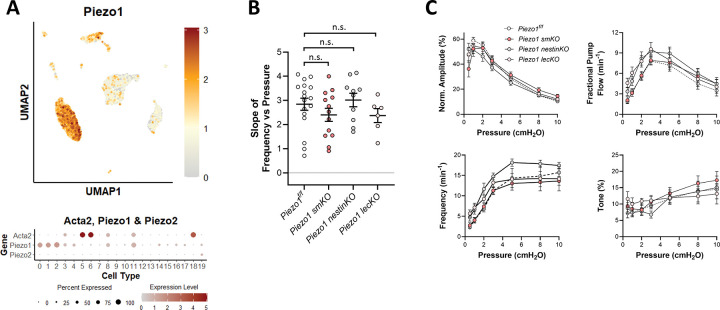
Consequences of *Piezo1* deletion. **A**) UMAP and bubble plots of *Piezo1 and Piezo2* expression in the various IALV cell clusters. **B**) Slope of F-P relationship for popliteal lymphatics from *Piezo1*^*f/f*^*, Piezo1 smKO*, *Piezo1 nestin KO*, *Piezo1 lecKO* mice. **C**) Summary plots of contraction parameters as a function of pressure for the 4 respective groups of vessels. *Piezo1*^*f/f*^ N=9, n=17; *Piezo1 smKO* N=7, n=13; *Piezo1 nestin KO* N=5, n=10; *Piezo1 lecKO* N=5, n=6.

PIEZO1 and PIEZO2 are two ion channels that satisfy the full set of criteria for true mechanosensitivity (Coste et al., 2010; Davis et al., 2023c). PIEZO2 is expressed primarily in neurons (Woo et al., 2014; Woo et al., 2015) whereas PIEZO1 is more widely expressed (Murthy et al., 2017). PIEZO1 is critically important for shear-stress induced Ca^2+^ signaling in endothelium (Li et al., 2014a), for lymphatic valve and vessel development (Nonomura et al., 2018; Choi et al., 2019; Choi et al., 2024) and for hypertension-dependent arterial wall remodeling (Retailleau et al., 2015). The role of PIEZO1 in the arterial myogenic response has not been systematically tested, except for a single report showing that SM-specific *Piezo1* deletion did not alter myogenic tone development in either caudal and rostral cerebellar arteries of mice [Suppl. Figs. 2–3 in (Retailleau et al., 2015)]. The possible involvement of PIEZO1 in lymphatic vessel contractility has not been reported. Our scRNAseq analysis revealed strong expression of *Piezo1* message in LECs but very weak expression in LMCs (Fig. 3A). *Piezo2* expression was barely detectable in either cell type. Popliteal lymphatic vessels from Myh11-CreER^T2^*;Piezo1*^*f/f*^ (*Piezo1 smKO*) and *Piezo1*^*f/f*^ control mice were subjected to the protocol for assessing pressure-induced chronotropy. However, the data revealed no significant difference in the F-P slope between *Piezo1 smKO* vessels and *Piezo1*^*f/f*^ control vessels (Fig. 3B), suggesting that PIEZO1 channels in LMCs are not required for pressure-induced lymphatic chronotropy. As a further test, we used Nestin-Cre to generate Nestin-Cre*;Piezo1*^*f/f*^ *(Piezo1 nestinKO)* mice with constitutive deletion of *Piezo1* both in smooth muscle and other cells expressing intermediate filaments. An analysis of vessel responses from *Piezo1 nestinKO* mice indicated that there was no significant impairment in pressure-induced chronotropy (Fig. 3B). Although we did not suspect the endothelium would be involved in pressure-induced chronotropy, we also generated and tested Prox1-CreER^T2^*;Piezo1*^*f/f*^ *(Piezo1 lecKO)* mice. As expected, there was no significant difference in the average F-P slope of vessels from *Piezo1 lecKO* mice compared to tamoxifen-treated *Piezo1*^*f/f*^ controls (Fig. 3B). Complete sets of contraction data for popliteal vessels from *Piezo1 smKO* are shown in Fig. 3C and revealed no significant differences in any of the lymphatic contractile parameters at any pressure compared to their floxed controls.

### Roles of TRPC6 and TRPC3.

**Figure 4.**
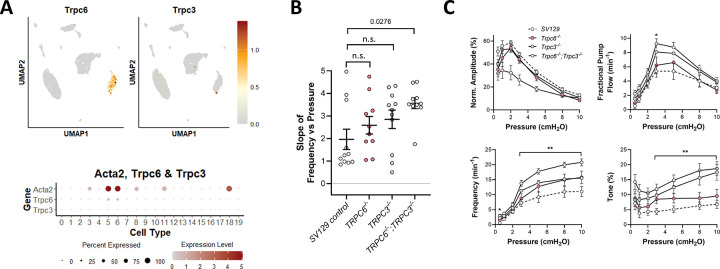
Consequences of *Trpc6* and/or *Trpc3* deletion. **A**) UMAP and bubble plots of *Trpc6 and Trpc3* expression in the various IALV cell clusters. **B**) Slope of F-P relationship for popliteal lymphatics from SV129 controls, *Trpc6*^−/−^*, Trpc3*^−/−^ *and Trpc6*^−/−^*;Trpc3*^−/−^ mice. **C**) Summary plots of contraction parameters as a function of pressure for the 4 respective groups of vessels. SV129 control N=8, n=11; *Trpc6*^−/−^ N=5, n=10; *Trpc3*^−/−^ N=7, n=11; *Trpc6*^−/−^*;Trpc3*^−/−^ N=5, n=10.

TRPC6 is thought to be a mechanosensitive cation channel whose activation underlies pressure-induced constriction in cerebral arteries (Welsh et al., 2002). However, studies of other arteries from *Trpc6*^−/−^ mice found either no differences from control vessels or even *enhanced* myogenic responsiveness (Dietrich et al., 2005; Schleifenbaum et al., 2014). Because global TRPC6 deletion results in compensatory upregulation of TRPC3 (Dietrich et al., 2005), a related TRPC family member with similar properties and which is also activated by DAG, it is possible that TRPC3 could compensate for loss of TRPC6. The expression patterns for TRPC6 and TRPC3, based on scRNAseq analyses, are shown in Fig. 4A. TRPC6 was expressed at modest levels in ~50% of LMCs and was almost exclusively confined to LMCs. TRPC3 was expressed at very low levels in only a few LMCs (from control mice). We tested the effects of global TRPC6 deletion (*Trpc6*^−/−^), global TRPC3 deletion (*Trpc3*^−/−^) and global deletion of both isoforms (*Trpc6*^−/−^*;Trpc3*^−/−^) on pressure-induced lymphatic chronotropy. As these mice were all generated in the SV129 background, control measurements were performed on popliteal vessels from SV129 mice, which showed a *lower* average F-P slope than WT (C57Bl/6) popliteal vessels. There were no significant differences in F-P slope between SV129 controls and *Trpc6*^−/−^ or *Trpc3*^−/−^ vessels, but the F-P slope of *Trpc6*^−/−^*;Trpc3*^−/−^ vessels was significantly *higher* than that of the SV129 controls (Fig. 4B). Complete sets of contraction data for *Trpc6*^−/−^*;Trpc3*^−/−^ vessels, compared to their *SV129* controls, are shown in Fig. 4C. *Trpc6*^−/−^*;Trpc3*^−/−^ double knock-out vessels had significantly impaired contraction amplitudes at several pressures and *increased* frequencies and *increased* levels of tone at most pressures. However, relevant to the main objective of this study, there were no significant *impairments* in pressure-induced chronotropy in vessels deficient in TRPC6, TRPC3 or both TRPC6 and TRPC3 (Fig. 4B–C).

### Role of TRPM4.

**Figure 5.**
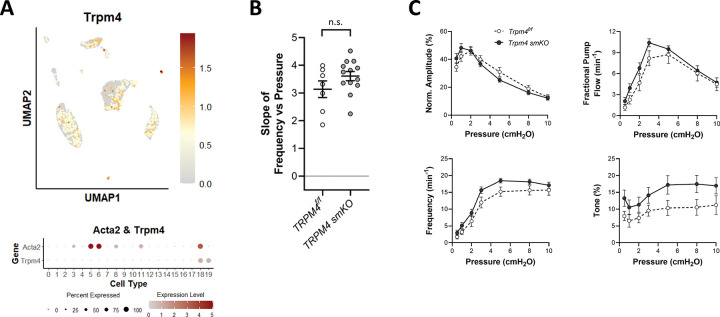
Consequences of *Trpm4* deletion. **A**) UMAP and bubble plots of *Trpm4* expression in the various IALV cell clusters. **B**) Slope of F-P relationship for popliteal lymphatics from *Trpm4*^*f/f*^ *and Trpm4 smKO* mice. **C**) Summary plots of contraction parameters as a function of pressure for the two respective groups of vessels. *Trpm4 smKO* N=6, n=13; *Trpm4*^*f/f*^ N=4, n=7.

Multiple studies have implicated TRPM4 channels in the arterial myogenic response (Gonzales & Earley, 2013; Earley & Brayden, 2015), but to date no published studies have examined the role of TRPM4 in lymphatic vessel contractility. scRNAseq analysis revealed that *Trpm4* was expressed at modest levels in only ~10% of LMCs, and in a subset of LECs, but at higher levels in some AdvCs (Fig. 5A). We tested the role of TRPM4 in pressure-induced lymphatic chronotropy by comparing the responses of Myh11-CreER^T2^*;Trpm4*^*f/f*^ vessels to *Trpm4*^*f/f*^ vessels. The lack of significant reductions in F-P slope of *Trpm4 smKO* vessels (Fig. 5B) suggests that TRPM4 is not required for pressure-induced lymphatic chronotropy. Contraction data comparing *Trpm4 smKO* vessels to *Trpm4*^*f/f*^ popliteal vessels are shown in Fig. 4C. There were no significant differences between the contraction parameters of *Trpm4 smKO* and *Trpm4*^*f/f*^ vessels at any pressure.

### Role of PKD2.

**Figure 6.**
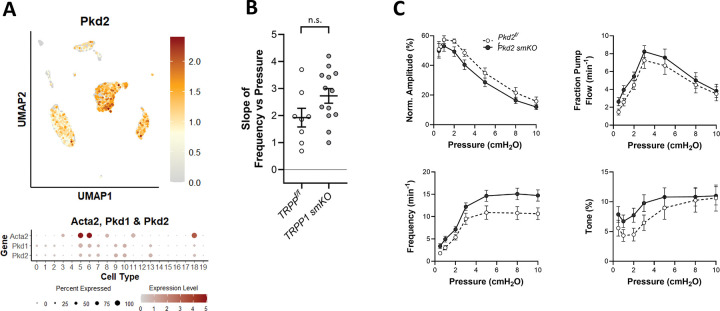
Consequences of *Pkd2* (TRPP1) deletion. UMAP and bubble plots of *Pkd2* expression in the various IALV cell clusters. **B**) Slope of F-P relationship for popliteal lymphatics from *Pkd2* ^*f/f*^ *and Pkd2 smKO* mice. **C**) Summary plots of contraction parameters as a function of pressure for the two respective groups of vessels. *Pkd2 smKO* N=6, n=13; *Pkd2* ^*f/f*^ N=4, n=8.

Studies by Jaggar and colleagues indicate that SM-specific deletion of PKD2 attenuates pressure-induced depolarization and myogenic tone in rat pial arteries (Narayanan et al., 2013) and blunts pressure-induced constriction in hindlimb arteries (Bulley et al., 2018). The roles of PKD2/2 channels in lymphatic vessel contractility are not known. scRNAseq analysis revealed modest *Pkd2* and *Pkd1* expression in LMCs and AdvCs (Fig. 6A). We tested the possible involvement of PKD2 channels in pressure-induced lymphatic chronotropy by comparing the responses of Myh11-CreER^T2^*;Pkd2*^*f/f*^ (*Pkd2 smKO*) vessels to *Pkd2*^*f/f*^ vessels (Fig. 6B). The lack of a significant reduction in the F-P slope for *Pkd2 smKO* vessels suggests that PKD2 is not required for pressure-induced lymphatic chronotropy. Contraction data comparing *Pkd2 smKO* vessels to *Pkd2*^*f/f*^ popliteal vessels are shown in Fig. 6C. There were no significant differences between the contraction parameters of *Pkd2 smKO* and *Pkd2*^*f/f*^ vessels at any pressure.

### Role of TRPV4.

**Figure 7.**
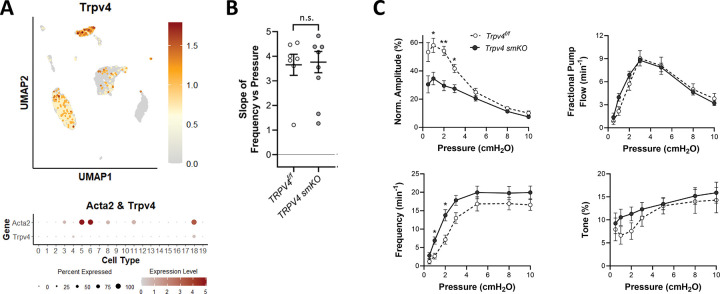
Consequences of *Trpv4* deletion. UMAP and bubble plots of *Trpv4* expression in the various IALV cell clusters. **B**) Slope of F-P relationship for popliteal lymphatics from *Trpv4*^*f/f*^ *and Trpv4 smKO* mice. **C**) Summary plots of contraction parameters as a function of pressure for the two respective groups of vessels. *TRPV4 smKO* N=5, n=10; *TRPV4*^*f/f*^ N=3, n=7.

In the blood vasculature, TPRV4 has been studied extensively in the context of Ca^2+^ influx pathways in endothelium (Chen & Sonkusare, 2020). However, TRPV4 is also expressed in VSM cells of some arteries (Mercado et al., 2014) and one report indicates that pharmacological inhibition of TRPV4 channels blunts pressure-induced VSM cell depolarization and myogenic vasoconstriction of preglomerular arteries from neonatal pigs (Soni et al., 2017). Our scRNAseq analysis revealed that TRPV4 was largely undetectable in LMCs but expressed at higher levels in almost all LECs, macrophages and some AdvCs (Fig. 7A). Nonetheless, we tested the role of TRPV4 channels in pressure-induced lymphatic chronotropy by comparing the responses of Myh11-CreER^T2^*;Trpv4*^*f/f*^ vessels to *Trpv4*^*f/f*^ vessels. The lack of a significant reduction in the F-P slope for *Trpv4 smKO* vessels suggests that TRPV4 is not required for pressure-induced lymphatic chronotropy (Fig. 7B). Contraction data comparing *Trpv4 smKO* and *Trpv4*^*f/f*^ popliteal vessels are shown in Fig. 7C. Contraction amplitude was impaired at several pressures in *Trpv4 smKO* vessels, suggesting some involvement of TRPV4 channels in control of contractile strength, possibly as a result of macrophage-released products (Schulz et al., 2025). Frequency was significantly different at two lower pressures in *Trpv4 smKO* vessels (Fig. 7C), where it was *elevated* rather than reduced. We also tested the F-P relationship in *Trpv4*^−/−^ (global KO) vessels and confirmed that there was no significant impairment in pressure-induced lymphatic chronotropy (average slope = 2.7 ± 0.2; n=18) compared to WT vessels (average slope = 2.8 ± 0.3; data not shown). Collectively, these results indicate that TRPV4 channels are not required for pressure-induced lymphatic chronotropy.

### Role of TRPV2.

**Figure 8.**
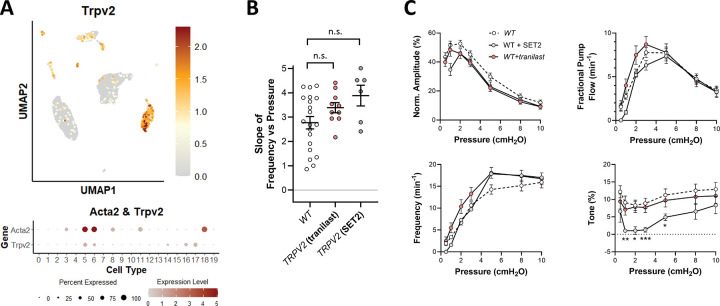
Consequences of Trpv2 inhibition. UMAP and bubble plots of *Trpv2* expression in the various IALV cell clusters. **B**) Slope of F-P relationship for popliteal lymphatics from WT mice with/without treatment with the TRPV2 inhibitors tranilast (10 μM) and SET2 (1 μM). **C**) Summary plots of contraction parameters as a function of pressure for the three respective groups of vessels. WT+tranilast N=5, n=10; WT+SET2 N=3, n=6.

TRPV2 is an osmosensitive cation channel (Nilius & Droogmans, 2001). Down-regulation of TRPV2 expression in mouse aortic VSM cells reduced the amplitude of cation currents activated by hypotonic swelling (Muraki et al., 2003). In another study, application of a hypotonic bath solution stimulated TRPV2-like whole-cell cation currents and Ca^2+^ influx in VSMCs isolated from rat retinal arteries—responses that were attenuated by the TRPV2 inhibitor tranilast (McGahon et al., 2016). Additionally, the administration of tranilast to rat retinal arteries resulted in loss of pre-existing myogenic tone whereas pre-incubation with a TRPV2 blocking antibody prevented the development of myogenic tone (McGahon et al., 2016). Our scRNAseq analysis revealed that TRPV2 was highly expressed in 63% of *Myh11*-expressing LMCs (Fig. 8A). As we did not have access to TRPV2 null or floxed mice, we tested the potential role of TRPV2 channels in pressure-induced lymphatic chronotropy by bath application of tranilast (100 μM) to WT popliteal lymphatic vessels. There was no significant reduction in the F-P slope for tranilast-treated vessels (Fig. 8B). We also tested the effects of SET2, a recently reported TRPV2 inhibitor with a lower IC_50_ (460 nM) than tranilast. The application of SET2 (1 μM) caused a transient cessation of spontaneous contractions for 2–5 min in 5 of 6 vessels, but the contractions returned within 10 min in the continued presence of SET2. Subsequent pressure steps revealed that SET2 did not significantly alter the F-P slope (Fig. 8B). Contraction data comparing WT and tranilast- and SET2-treated WT popliteal vessels are shown in Figs. 8C. These results suggest that TRPV2 is not required for pressure-induced lymphatic chronotropy.

### Role of ENaC.

**Figure 9.**
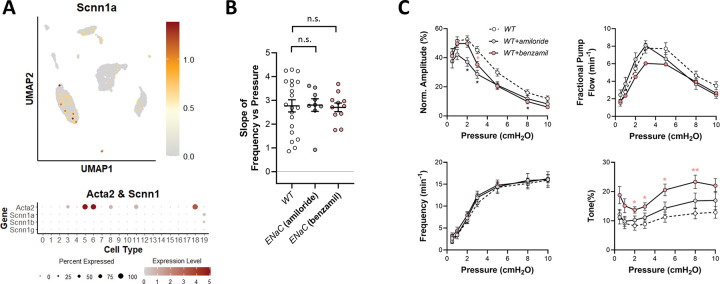
Consequences of ENaC inhibition. UMAP and bubble plots of *ENaC* subsunit expression in the various IALV cell clusters. **B**) Slope of F-P relationship for popliteal lymphatics from WT mice with/without treatment with the ENaC inhibitors amiloride (5 μM) and benzamil (1 μM). WT N=15, n=20, WT+amiloride N=5, n=9; WT+benzamil N=6, n=11.

The epithelial Na^+^ channel (ENaC), comprised of a heterotrimer under the expression of the genes *Scnn1a*, *Scnn1b*, *Scnn1g*, *Scnn1d*, is expressed in both the endothelial and smooth muscle layers of arteries. αENaC is the principal isoform in endothelium and studies of native epithelial or heterologous channels reconstituted into bilayers suggest that αENaC is intrinsically mechanosensitive (Price et al., 2000; Benos, 2004). Work from the Drummond laboratory suggests that the βENaC isoform is expressed in VSM cells and plays a critically important role in myogenic constriction (Drummond, 2012). However, our scRNAseq analysis revealed almost no expression of αENaC subunits in LMCs and only weak expression in the LEC population (Fig. 9A); βENaC was essentially undetectable. Nevertheless, we tested the possible role of ENaC in pressure-induced lymphatic chronotropy by administering two widely used ENaC inhibitors, amiloride and benzamil, to WT popliteal lymphatic vessels. The data in Fig. 9B show that neither amiloride nor benzamil, at concentrations known to inhibit arterial myogenic constriction [5 and 1 μM, respectively (Jernigan & Drummond, 2006)], caused a significant impairment in pressure-induced lymphatic chronotropy. Contraction data for amiloride- and benzamil-treated WT popliteal vessels are shown in Fig. 9C. Both ENaC inhibitors tended to impair contraction amplitude to some degree (significant only for amiloride), with benzamil also significantly increasing tone at almost all pressure levels. However, the F-P relationships were nearly identical between WT and amiloride- or benzamil-treated vessels, reinforcing the conclusion that ENaC is not involved in pressure-induced lymphatic chronotropy.

### Role of NCX1.

**Figure 10.**
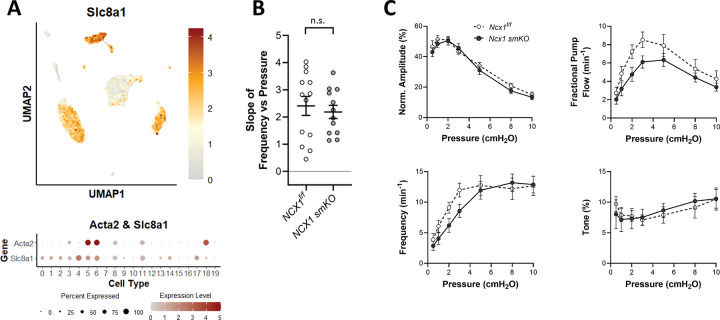
Consequences of *Slc8a1* (NCX1) deletion. UMAP and bubble plots of *Slc8a1* expression in the various IALV cell clusters. **B**) Slope of F-P relationship for popliteal lymphatics from *Slc8a1*^*f/f*^ *and Slc8a1 smKO* mice. **C**) Summary plots of contraction parameters as a function of pressure for the two respective groups of vessels. *Slc8a1*^*f/f*^ N=7, n=12; *Slc8a1 smKO* N=7, n=13.

The sodium-calcium exchanger (NCX) has a well-documented role in controlling the pacemaker activity of sinoatrial nodal cells, where it couples SR calcium oscillations, termed local calcium releases (LCRs) caused by calcium movement through ryanodine channels (Vinogradova et al., 2004; Vinogradova et al., 2005), to AP ignition. LCRs may occur throughout diastole (Monfredi et al., 2013), but their role is particularly critical in late diastole where LCRs activate NCX and the ensuing addition of inward current, and positive feedback of further calcium influx through L-type channels, provides sufficient depolarization to cross the voltage threshold for initiating AP firing (Bogdanov et al., 2001; Lyashkov et al., 2018b). This process is possibly analogous to the electrophysiological pacemaking mechanism in LMCs. scRNAseq analysis revealed strong expression of *Slc8a*, which encodes NCX1, in >80% of both LMCs and expression was also noted in LECs and some immune cells (Fig. 10A). We tested the role of NCX1 in pressure-induced chronotropy by comparing the responses of Myh11-CreER^T2^*;Slc8a1*^*f/f*^ (*Slc8a1 smKO*) and *Slc8a1*^*f/f*^ vessels. The lack of a significant reduction in the F-P slope for *Slc8a1 smKO* vessels suggests that NCX1 is not required for pressure-induced lymphatic chronotropy (Fig. 10B). Contraction data for *Slc8a1 smKO* and *Slc8a1*^*f/f*^ popliteal vessels are shown in Fig. 10C and reveal no significant differences between the contraction parameters of *Slc8a1 smKO* and *Slc8a1*^*f/f*^ vessels.

### Role of HCN.

**Figure 11.**
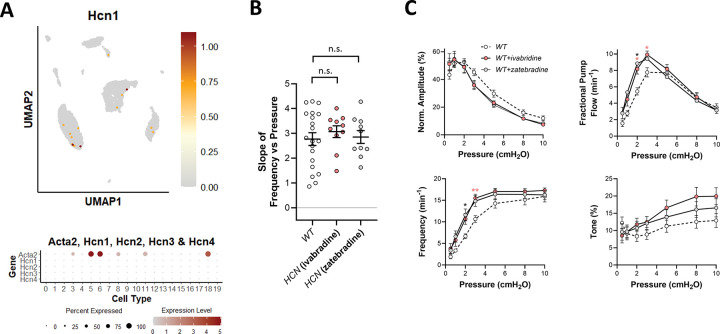
Consequences of HCN inhibition. UMAP and bubble plots of *Hcn gene* family expression in the various IALV cell clusters. **B**) Slope of F-P relationship for popliteal lymphatics from WT mice with/without treatment with the HCN inhibitors ivabradine (3 μM) and zatebradine (3 μM). WT N=15, n=20, WT+zatebradine. N=5, n=10; WT+ivabradine N=5, n=10.

HCN (Hyperpolarization-activated cyclic-nucleotide-gated) channels contribute an inward “funny” current (I_f_) to the diastolic pacemaking potential of sinoatrial node cells (Accili et al., 2002) and are also involved in the intrinsic rhythmicity of bladder and uterine smooth muscle (Alotaibi et al., 2017; Mader et al., 2018). In LMCs they could potentially contribute to diastolic depolarization. The HCN blockers CsCl, ZD7288 and ivabradine all lower the rate of spontaneous contractions of lymphatic vessels in rat diaphragm (Negrini et al., 2016), but inhibition requires substantially higher concentrations than the documented IC_50_ values for HCN channel blockade [see discussion in (Davis & Zawieja, 2024)]. In contrast, all three inhibitors *increase* the spontaneous contraction frequency of human lymphatic vessels at concentrations near or slightly higher than the IC_50_ values for HCN inhibition (Majgaard et al., 2022), suggesting off-target effects in some lymphatic vessels. Our scRNAseq analysis revealed essentially no expression of any of the four HCN isoforms in LMCs (Fig. 11A), however it is possible the expression is too low to detect without deep sequencing. Due to the proven role of HCN channels in cardiac pacemaking, we tested the role of HCN channels in pressure-induced lymphatic chronotropy of WT mouse popliteal lymphatics using the HCN inhibitor ivabradine (at a concentration of 3 μM, which is slightly higher than the IC_50_ of 2.5 μM), and the more effective inhibitor, zatebradine, at a concentration of 3 μM (IC_50_ = 480 nM). The lack of significant reductions in the F-P slope for ivabradine- or zatebradine-treated WT vessels suggests that HCN channels are not required for pressure-induced lymphatic chronotropy (Fig. 11B). Contraction data for ivabradine- and zatebradine-treated WT vessels are shown in Fig. 11C and reveal no substantial differences from WT vessels, except for a single pressure in which each HCN channel inhibitor actually *increased* the contraction frequency. Collectively, these results recapitulate the findings of Majgaard et al (Majgaard et al., 2022) and reinforce the conclusion that HCN channels are not required for pressure-induced lymphatic chronotropy.

### Role of Kv7.4.

**Figure 12.**
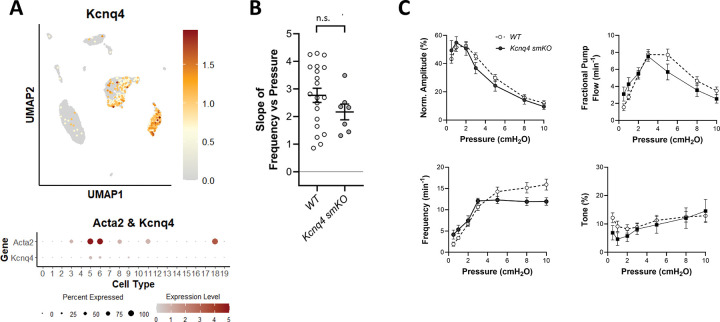
Consequences of *Kcnq1* deletion. UMAP and bubble plots of *Kcnq4* expression in the various IALV cell clusters. **B**) Slope of F-P relationship for popliteal lymphatics from WT *and Kcnq4 smKO* mice. **C**) Summary plots of contraction parameters as a function of pressure for the two respective groups of vessels. *Kcnq4 smKO* N=5, n=7.

Kv7 channels are activated at more negative membrane potentials than Kv1-family delayed rectifier K^+^ channels and thus are implicated in control of VSMC excitability near the resting membrane potential (Yeung & Greenwood, 2005; Zhong et al., 2010; Mani & Byron, 2011). The Kv7 blocker XE991 enhances myogenic tone in isolated mesenteric and renal arteries and hypotonic swelling suppresses an XE991-sensitive Kv current in patch-clamped VSM cells (Schleifenbaum et al., 2014). The collective implication from these studies is that mechanosensitive *inhibition* of Kv7.4 may increase VSM excitability. The effects of Kv7 channel inhibition on lymphatic function are unknown but the pan-Kv7 channel inhibitors linopirdine and XE991 cause concentration-dependent increases in lymphatic contraction frequency while the Kv7 activator ML-277 inhibits spontaneous lymphatic contractions (our unpublished observations in rat mesenteric lymphatic vessels). Our scRNAseq analysis revealed moderately strong expression of *Kcnq4* in >50% LMCs and in some AdvCs (Fig. 12A). We tested whether pressure-induced lymphatic chronotropy was altered in Myh11-CreER^T2^*;Kcnq4*^*f/f*^ (*Kcnq4 smKO)* mice. However, the lack of a significant reduction in the F-P slope for *Kcnq4 smKO* vessels suggests that Kv7.4 channels are not required for pressure-induced lymphatic chronotropy (Fig. 12B). Contraction data for *Kcnq4 smKO* and *WT* vessels are compared in Fig. 12C. There were no significant differences in any of the parameters at any pressure.

### Role of Kir channels.

**Figure 13.**
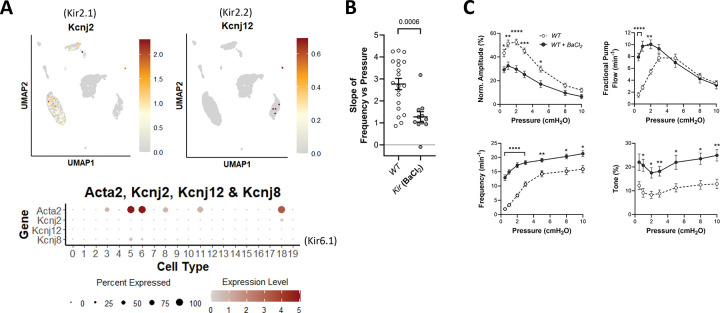
Consequences of Kir2.x inhibition. UMAP and bubble plots of *Kcnj2* (Kir2.1*), Kcnj12 (*Kir2.2*)* and *Kcnj8* (*Kir6.1)* expression in various IALV cell clusters. **B**) Slope of F-P relationship for popliteal lymphatics from WT mice with/without treatment with the Kir2 inhibitor BaCl_2_ (100 μM). WT N=15, n=20, WT+BaCl_2_ N=4, n=11.

Inwardly rectifying K^+^ channels, including Kir2.1 and Kir2.2 which are encoded by *Kcnj2* and *Kcnj12* respectively, have been implicated in shear-stress-induced responses of arterial endothelium (Ahn et al., 2017) and swelling-induced currents in arterial smooth muscle cells (Sancho et al., 2019). However, their potential roles in controlling LMC membrane potential and pressure-induced lymphatic chronotropy are unknown. Our scRNAseq analysis revealed modest expression of *Kcnj8* (Kir6.1), an inward rectifier which forms the pore forming unit of the K_ATP_ channel, in >50% LMCs (Fig. 13A), but very little expression of *Kcnj2* or *Kcnj12* in LMCs. We previously showed that deletion of *Kcnj8* does not alter the F-P relationship in popliteal lymphatics (Davis et al., 2020). Here, we tested the effects of BaCl_2_, a selective inhibitor of Kir2.1/2.2 at a concentration of 100 μM (Longden et al., 2017; Sancho et al., 2017; Sancho et al., 2019), on the F-P relationship of WT popliteal lymphatic vessels. Kir inhibition resulted in a significantly reduced slope of the F-P relationship compared to WT vessels (Fig. 13B), but the consistent effect of Ba^2+^ was to flatten the slope of the F-P relationship by substantially *increasing* the contraction frequency at low pressures while only slightly *increasing* the contraction frequency at elevated pressures (Fig. 13C). This contrasts with the effect of ANO1 inhibition or deletion: in the absence of ANO1 activity, the contraction frequency was significantly reduced at all pressures, particularly at low pressures (Fig. 2C). Thus, the inhibition of Kir channels does impair pressure-induced chronotropy but does so by differentially *elevating* contraction frequency as a function of pressure.

### Roles of L- and T-type Ca^2+^ channels.

**Figure 14.**
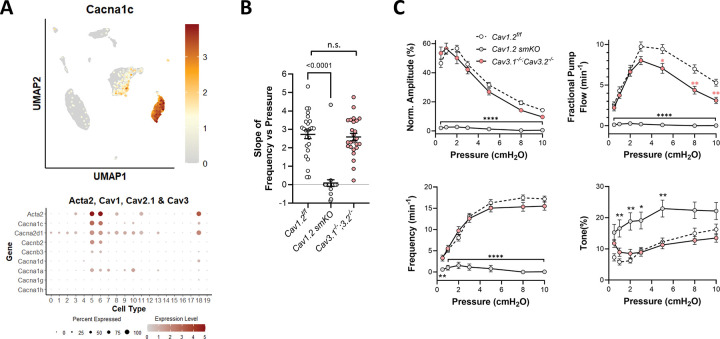
Consequences of *Cacna1c* (Cav1.2) and *Cacna1g/Cacna1h* (Cav3.1/3.2) deletion. UMAP and bubble plots of the expression of voltage-gated Ca^2+^ channels and their accessory subunits in the various IALV cell clusters. **B**) Slope of F-P relationship for popliteal lymphatics from *Cacan1c*^*f/f*^*, Cacna1c smKO* and *Cacna1g*^−/−^*;Cacna1h*^−/−^ mice. **C**) Summary plots of contraction parameters as a function of pressure for the three respective groups of vessels. *Cacna1c*: alpha-1c, *Cacna2d1*: α2/δ1, *Cacnb2*: β2, *Cacnb3*: β3, *Cacna1d*: alpha-1d (Ca_v_1.3), *Cacna1a*: Ca_v_2.1 (P/Q), *Cacna1g*: Ca_v_3.1, *Cacna1h*: Ca_v_3.2. *Cacna1c*^*f/f*^ N=10, n=20; *Cacna1c smKO* N=14, n=24; *Cacna1g*^−/−^*;Cacna1h*^−/−^ N=16, n=25.

The L-type, voltage-gated Ca^2+^ channel (Cav1.2) carries the majority of current contributing to the action potential in LMCs (van Helden, 1993; To et al., 2020). scRNAseq analysis confirmed that >90% of LMCs expressed message for the pore forming subunit of Cav1.2 (*Cacna1c*), along with other accessory subunits of the channel responsible for either gating characteristics (α2δ) or membrane targeting (β) (Fig. 14A). SM-specific knockout of *Cacna1*, using either the Myh11-CreER^T2^ or Itga8-CreER^T2^, eliminated essentially all propulsive contractions from popliteal lymphatic vessels (Warthi et al., 2022; Davis et al., 2023d). Residual activity in some vessels was limited to small, localized diameter oscillations (<5 μm in amplitude) with irregular frequencies that were largely independent of pressure. As expected, the slope of the F-P relationship for *Cacna1c smKO* vessels was significantly reduced compared to *Cacna1c*^*f/f*^ control vessels (Fig. 14B).

The T-type Ca^2+^ channel (Cav3.x) is critical for pacemaking in SA node (Lyashkov et al., 2018a), GI smooth muscle (Sanders, 2019) and renal pelvis smooth muscle (Grainger et al., 2022). Two Cav3 isoforms are expressed in LMCs: *Cacna1g* and *Cacna1h,* encoding Cav3.1 and Cav3.2 respectively (Lee et al., 2014; To et al., 2020). One report suggests that Cav3 channels are selectively involved in regulating the contraction frequency (but not amplitude) of rat mesenteric lymphatic collectors (Lee et al., 2014). We previously tested the roles of Cav3.1 and Cav3.2 in mouse lymphatic collectors and isolated LMCs after generating *Cacna1g*^−/−^*;Cacna1h*^−/−^ double KO mice (To et al., 2020). Here, we analyzed the slope of the F-P relationship in popliteal lymphatics from *Cacna1g*^−/−^*;Cacna1h*^−/−^ mice but found no significant differences from either WT or *Cav1.2*^*f/f*^ control vessels (Fig. 14B), indicating that Cav3 channels are not involved in determining the F-P relationship.

### Upstream pathways mediating pressure-induced chronotropy

Having established that ANO1, out of all the cation and K^+^ channels tested, is the only current mediating pressure-induced lymphatic chronotropy, we then turned to an investigation of mechanosensitive pathways upstream from ANO1 channel activation.

#### Role of IP_3_R in pressure-induced chronotropy.

**Figure 15.**
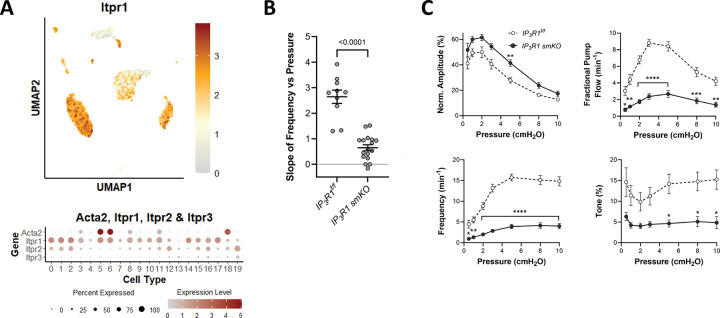
Consequences of *Itpr1* (IP_3_R1) deletion. UMAP and bubble plots of *Ip3r1* expression in the various IALV cell clusters. **B**) Slope of F-P relationship for popliteal lymphatics from *Itpr1*^*f/f*^ *and Itpr1 smKO* mice. **C**) Summary plots of contraction parameters as a function of pressure for the two respective groups of vessels. *Itpr1 smKO* N=5, n=8; *Itpr1*^*f/f*^ N=5, n=10.

Calcium release as a consequence of IP_3_ generation is known to activate a number of ion channels and other proteins, including ANO1. We recently showed that genetic deletion of *Itpr1* (encoding IP_3_R1) from mouse IALVs results in a reduction in spontaneous contraction frequency and a blunting of pressure-induced chronotropy similar to what is observed after *Ano1* deletion (Zawieja et al., 2023). scRNAseq analysis confirmed expression of *Itpr1* in >50% of LMCs (Fig. 15A). Here, we examined the consequences of *Itpr1* deletion in popliteal lymphatics by comparing the F-P relationship for vessels from Myh11-CreER^T2^*;Itpr1*^*f/f*^ (*Itpr1 smKO*) and *Itpr1*^*f/f*^ mice. SM-specific deletion of *Itpr1* resulted in a significant reduction in the slope of the F-P relationship from 2.6 ± 0.3 to 0.6 ± 0.1 (Fig. 15B). Contraction data for *Itpr1 smKO* and *Itpr1*^*f/f*^ control vessels are shown in Fig. 15C. The most striking features are 1) the significant reductions in frequency at all pressures and 2) a significant reduction in tone at elevated pressures in *Itpr1 smKO* vessels. The latter effect contrasts with the *increases* in tone observed after ANO1 inhibition or deletion (Fig. 2C). Collectively, these results show that IP_3_R1 is critically important for pressure-induced lymphatic chronotropy in popliteal collecting vessels in much the same way it was previously documented in IALVs. Deletion of *Itpr1* in LMCs produces an effect on pressure-induced chronotropy similar to that of ANO1 deletion.

#### Role of GNAQ/GNA11 in pressure-induced chronotropy.

**Figure 16.**
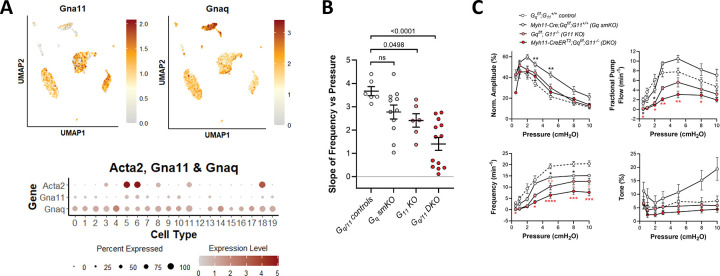
Consequences of *Gnaq* and/or *Gna11* deletion. UMAP and bubble plots of *Gnaq* and *Gna11* expression in the various IALV cell clusters. **B**) Slope of F-P relationship for popliteal lymphatics from *Gnaq*^*f/f*^*;Gna11*^+/+^, Myh11-CreER^T2^*;Gnaq*^*f/f*^ (Gnaq smKO*), Gna11*^−/−^ *and* Myh11-CreER^T2^*;Gnaq*^*f/f*^*;Gna11*^−/−^ *DKO* mice. **C**) Summary plots of contraction parameters as a function of pressure for the four respective groups of vessels. *Gnaq*^*f/f*^*;Gna11*^+/+^ control N=3, n=6; *Gnaq smKO* N=4, n=8; *Gna11*^−/−^ N=3, n=6; Myh11-CreER^T2^*;Gnaq*^*f/f*^*;Gna11*^−/−^ N=8, n=13.

Evidence from multiple studies specifically points to GNAQ/GNA11-coupled GPCRs as the upstream mechanosensing elements of myogenic constriction in arteries (Mederos y Schnitzler et al., 2008; Blodow et al., 2014; Harraz et al., 2014; Li et al., 2014b; Schleifenbaum et al., 2014; Pires et al., 2017; Bjorling et al., 2018). Mechanosensitive activation of one or more GPCRs independent of ligand binding (Zou et al., 2004; Erdogmus et al., 2019) could account for pressure-induced increases in IP_3_ levels in LMCs. Our scRNAseq analysis revealed strong expression of *Gnaq* and *Gna11* in ~75% of LMCs, LECs, and AdvCs as well as very strong expression of *Gnaq* in some immune cell clusters (Fig. 16A). We tested the effects of SM-specific *Gnaq* knock out (Myh11-CreER^T2^*;Gnaq*^*f/f*^), global *Gna11* knock out (*Gna11*^−/−^) and *Gnaq*/*Gna11* double knock out (Myh11-CreER^T2^*;Gnaq*^*f/f*^*;Gna11*^−/−^) on pressure-induced lymphatic chronotropy. There was a trend for *Gnaq smKO* vessels to have a reduced slope in the F-P relationship in comparison to *Gnaq*^*f/f*^*;Gna11*^+/+^ control vessels, but it did not reach statistical significance (p=0.1296). However, global deletion of *Gna11* significantly reduced the slope of the F-P relationship and the reduction was even more significant and pronounced in *Gnaq/Gna11* double KO vessels (Fig. 16B). However, even combined knock out of both proteins did not cause an attenuation of pressure-induced chronotropy that was comparable to that observed after SM-specific *Itpr1* or *Ano1* deletion, particularly at pressures > 2 cmH_2_O (compare Fig. 16B to Figs. 2B, 15B). Contraction data for *Gnaq smKO*, *Gna11*^−/−^ and *Gna*_*q*_*/Gna*_*11*_ double KO vessels, compared to tamoxifen-treated *Gnaq*^*f/f*^*;Gna11*^+/+^ controls, are shown in Fig. 16C. The full F-P relationships for each genotype showed significant reductions in frequency at a few pressures for *Gnaq smKO* vessels compared to *Gnaq*^*f/f*^*;Gna11*^+/+^ controls, significant differences for *Gna11*^−/−^ vessels at more pressures and significant differences at even more pressures for *Gnaq*/*Gna11* DKO vessels. Collectively, these results suggest that both GNAQ and GNA11-coupled G-proteins are involved in pressure-induced lymphatic chronotropy, with G11 being more important. However, the escape from inhibition at higher pressures points to the possible involvement of other mechanisms activating phospholipase(s) to generate IP_3_.

#### Role of GNA12/GNA13 in pressure-induced chronotropy.

**Figure 17.**
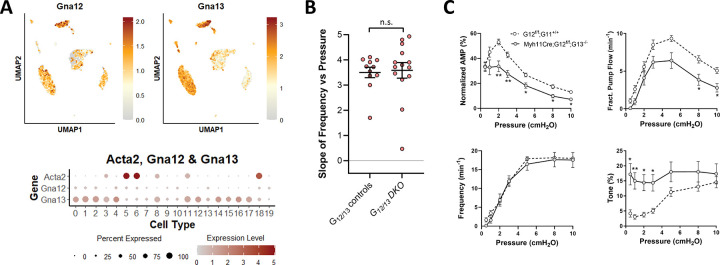
Consequences of *Gna12*/*Gna13* deletion. UMAP and bubble plots of *Gna12* and *Gna13* expression in the various IALV cell clusters. **B**) Slope of F-P relationship for popliteal lymphatics from *Gna12*^*f/f*^*;Gna13*^+/+^ *and* Myh11-CreER^T2^*;Gna12*^*f/f*^*;Gna13*^−/−^ *DKO* mice. **C**) Summary plots of contraction parameters as a function of pressure for the two respective groups of vessels. *Gna12*^*f/f*^*;Gna13*^+/+^ controls N=5, n=10; Myh11-CreER^T2^*;Gna12*^*f/f*^*;Gna13*^−/−^ N=7, N=15.

Chennupati et al. (Chennupati et al., 2019) made the surprising finding that GNA12/GNA13-coupled GPCRs, rather than GNAQ/GNA11-coupled GPCRs, mediated myogenic constrictions in mesenteric and cerebral arteries of mice. GNA12/GNA13-coupled GPCRs are known to regulate contraction through Rho kinase (McDuffie et al., 2024) and one study of rat mesenteric lymphatics suggests that RhoK is essential for phasic lymphatic contractions (Kurtz et al., 2014). We investigated the effects of deleting both *Gna12* and *Gna13* from LMCs (Myh11-Cre^T2^*;Gna12*^*f/f*^*;Gna13*^−/−^ mice) on the F-P relationship of popliteal lymphatics. In the *Gna12*/*Gna13* double knock out, contraction amplitude was significantly blunted and tone was significantly elevated at several pressures, but the F-P relationship was nearly identical to that of vessels from control *Gna12*^*f/f*^*;Gna13*^+/+^ mice (Fig. 17B–C).

#### Role of AT_1_R in pressure-induced chronotropy.

**Figure 18.**
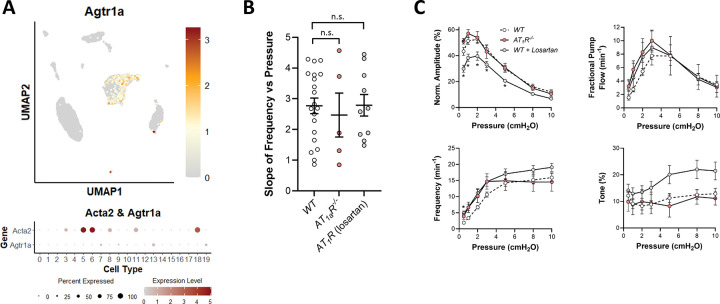
Consequences of *Agtr1* (AT_1_R) deletion/inhibition. UMAP and bubble plots of *Agtr1a* expression in the various IALV cell clusters. **B**) Slope of F-P relationship for popliteal lymphatics from WT mice with/without treatment with the AT_1_R inhibitor losartan (10 μM) and *Agtr1a*^−/−^ vessels. WT+losartan N=5, n=10; *Agtr1a*^−/−^ N=3, n=6.

In many arteries, AT_1_R is one of the predominantly expressed GPCRs and deletion of *Agtr1* results in loss or impairment of myogenic constriction in several types of arteries (Mederos y Schnitzler et al., 2008; Storch et al., 2012; Blodow et al., 2014; Schleifenbaum et al., 2014; Storch et al., 2015; Pires et al., 2017; Yamasaki et al., 2020). Selective deletion of the AT_1_R isoform (AT_1a_R or AT_1b_R) is sufficient to abrogate myogenic constriction in some arteries (Schleifenbaum et al., 2014; Pires et al., 2017). However, our scRNAseq analysis revealed almost no *Agtr1a* expression in LMCs, except in one possible subcluster (Fig. 18A). Pressure-induced chronotropy was measured in popliteal vessels from *Agtr1a*^−/−^ mice and in vessels from WT mice treated with the AT_1_R inhibitor losartan, which blocks both AT_1a_R and AT_1b_R. We used a concentration of losartan (10 μM) known to be sufficient to block myogenic constriction in several types of arteries (Blodow et al., 2014; Jackson & Boerman, 2017; Yamasaki et al., 2020). However, neither inhibition of AT_1_R nor deletion of *Agtr1a* impaired pressure-induced chronotropy, as evident by the absence of significant reductions in slope of the F-P relationship (Fig. 18B). Contraction data for these three groups are shown in Fig. 18C. Losartan treatment caused a slight impairment of contraction amplitude at several pressures but neither AT_1_R blockade nor *Agtr1a* deletion significantly inhibited the F-P relationship, suggesting that AT_1_R is not required for pressure-induced lymphatic chronotropy.

## Discussion

### Summary.

**Figure 19.**
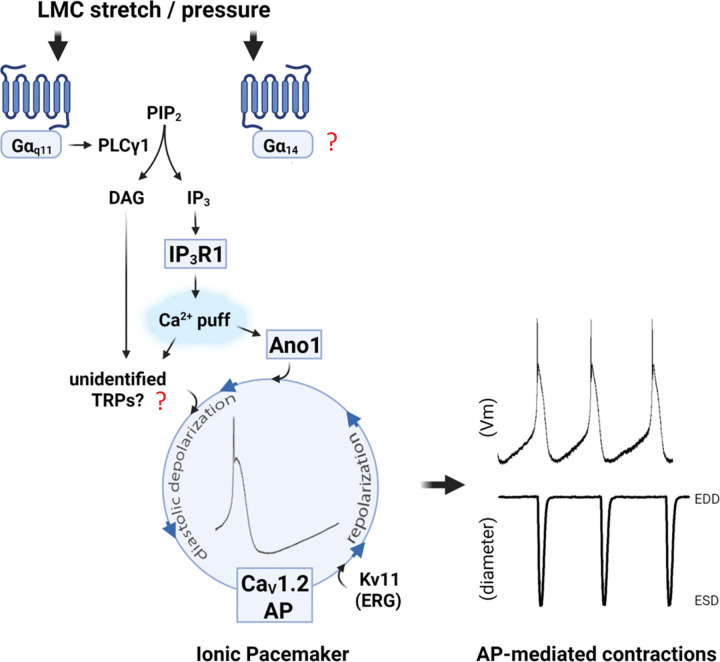
Proposed mechanotransduction mechanism underlying pressure-induced chronotropy. Summary diagram depicting the primary signaling pathway through which changes in pressure regulate the frequency of the ionic pacemaker. GNAQ/GNA11-coupled GPCRs are the primary, but not exclusive mechanosensor. ANO1 is the primary, but not exclusive effector. Kv11 provides at least one source of repolarizing signal (Kim et al., 2023).

The major goal of this study was to investigate the mechanistic basis of the F-P relationship in lymphatic muscle. The primary means by which lymphatic pumping is matched to excess filling of the interstitium is pressure-induced chronotropy—a >10-fold or more increase in contraction frequency with over the physiological pressure range (Davis et al., 2025). Because little was previously known regarding the mechanisms of mechanosensitivity underlying pressure-induced lymphatic chronotropy, we took cues from a well-studied mechanosensitive mechanism in vascular smooth muscle—the arterial myogenic response. In both arteries and collecting lymphatic vessels (Davis et al., 2009; Davis et al., 2023c), pressure elevation leads to vascular muscle cell depolarization and constriction, reflecting the activation of a depolarizing current that triggers Ca^2+^ entry through the opening of L-type, voltage-gated Ca^2+^ channels. Several ionic mechanisms have been previously implicated in this process for arterial smooth muscle, specifically the activation of TRPC6, TRPM4, PKD2, TRPV2 and ENaC cation channels (Welsh et al., 2002; Earley et al., 2004a; Jernigan & Drummond, 2005; Sharif-Naeini et al., 2009; McGahon et al., 2016), with supporting evidence for each obtained from gene knockdown / knockout studies. Additional ion channels may also be involved, including the activation of channels promoting Cl^−^ efflux (Boedtkjer et al., 2008) and the inhibition of constitutively active K^+^ channels (Sancho et al., 2019), but evidence for those is based largely on the use of pharmacological inhibitors and therefore subject to concerns about off-target effects (Maroto et al., 2005; Dietrich et al., 2007; Gottlieb et al., 2008; Sancho et al., 2019). In the present study, we primarily used genetic knock out approaches to test the possible roles of the major ion channels contributing to the mechanosensitive chronotropy of lymphatic vessels from the mouse. Our results strongly support the conclusion that pressure-induced lymphatic chronotropy does *not* involve acute activation of any of the putative mechanosensitive (and/or second-messenger gated) cation channels previously implicated in myogenic constriction (TRPC6, TRPM4, PKD2, TRPV2, ENaC), nor does it require PIEZO1, the bona fide mechanosensitive cation channel implicated in other aspects of mechanotransduction in the cardiovascular system [see references in (Davis et al., 2023c)]. Rather, our results align with evidence from related studies of arterial smooth muscle showing that mechanotransduction of stretch/pressure is mediated by GNAQ/GNA11-coupled GPCRs, leading to downstream activation of second messenger-gated channels. In the case of LMCs, mechano-activation of GNAQ/GNA11-coupled GPCRs promotes IP_3_ production that triggers Ca^2+^ release from stores through IP_3_R1; Ca^2+^ then activates ANO1 Cl^−^ channels, which accelerate the rate of spontaneous diastolic depolarization, increasing the firing rate of action potentials that control phasic lymphatic contractions (Fig. 19). Repolarization is controlled in part by Kv channels (Cotton et al., 1997), including Kv11 channels (Kim et al., 2023). As summarized in Table 1, knockout of each element in the GNAQ/GNA11- IP_3_-IP_3_R1/Ca^2+^-ANO1-Cav1.2 pathway (and only those elements out of all that were tested, except *Kcnj2/Kcnj12*, which can be alternatively explained) abrogated or significantly attenuated pressure-induced chronotropy. Mechanosensitive regulation of ANO1 in this context is likely mediated by Ca^2+^ release from stores rather than possible direct (Ca^2+^-independent) mechanosensitivity of ANO1 (Zou et al., 2025), because the similar degree of attenuation in the F-P relationship with *Iptr1* knockout as with *Ano1* smKO knock out shown here points to the involvement of an upstream mechanosensor.

### Possible involvement of other G-coupled proteins.

One inconsistency with the scheme suggested in Table 1 is that combined knock out of *Gnaq/Gna11* did not cause an attenuation of pressure-induced chronotropy comparable to that observed after SM-specific *Itpr1* or *Ano1* deletion, particularly at pressures > 2 cmH_2_O (compare Fig. 16B to Figs. 2B, 15B). A possible explanation is that other G-coupled proteins are involved in mechanosensitive regulation of the GPCR-IP_3_R1-Ca^2+^-ANO1 signaling in LMCs. Indeed, two other G protein-coupled effectors, *Gna14* and *Gna15* in the GNAQ family, could also be activated downstream of pressure-induced GPCR activation and potentially mediate IP_3_/DAG production. We have included *Gna14* (*Gna14* is expressed in 28% of LMCs in our dataset) in the summary scheme shown in Fig. 19 but its role in IP_3_ production and ANO1 activation remains to be investigated. Even so, inclusion of other G protein-coupled effectors would still fail to account for the residual pacemaking observed after apparently complete deletion of ANO1. We use the phrase “apparently complete” because incomplete recombination by the inducible Myh11-CreER^T2^ remains a possibility. Although we could not detect residual message for *Ano1* in Myh11-CreER^T2^*;Ano1*^*f/f*^ mice after tamoxifen treatment (Fig. 3B), that message was measured in LMCs digested from IALVs inguinal-axillary lymphatic vessels (chosen because of a higher cell yield), whereas the F-P relationship was measured in popliteal lymphatic vessels (chosen because of the greater dynamic range in F-P).

We also investigated the possible role of GNA12/GNA13-coupled GPCRs in the F-P relationship, based on a study showing that GNA12/GNA13, rather than GNAQ/GNA11, is required for myogenic constriction in arterial smooth muscle (Chennupati et al., 2019) and a paper showing that RhoK (known to be activated downstream from GNA12/GNA13 activation) is critical for phasic lymphatic contractions and associated phasic LMC Ca^2+^ signaling (Kurtz et al., 2014). However, *Gna12/Gna13* double knock out resulted in no significant change in the F-P slope (Fig. 17B), and there were no differences in frequency at any pressure compared to *Gna12*^f/f^*; Gna13*^+/+^ control vessels (Fig. 17C).

### Roles for ion channels in other aspects of LMC contractile function.

Originally, we hypothesized that an ion channel (other than ANO1) was involved in LMC pacemaking. If IP_3_ production in LMCs is enhanced after pressure elevation, as it is in arterial smooth muscle (Narayanan et al., 1994; Mederos y Schnitzler et al., 2008), then concomitant increases in DAG, the other product of PIP_2_ hydrolysis, would also be predicted to occur, and DAG should then activate TRP6C and related channels. However, if this occurs in popliteal lymphatic vessels, the activation of those channels apparently does not significantly influence the F-P relationship (Fig. 4). Likewise, the Ca^2+^ increase resulting from mechanosensitive activation of the GNAQ/GNA11-IP_3_-IP_3_R1 signaling axis would potentially activate TRPM4 and/or PKD2 (assuming those channels are localized to the appropriate LMC microdomains), but we find that their deletion does not alter the F-P relationship (Figs. 5–6). Because TRPM4, PKD2 and PIEZO1 expression levels are very low in LMCs (Figs. 3A,4A, 5A), further activation of those channels may have little effect. It is possible that another unidentified ion channel could be mediating the pressure-driven frequency changes at higher pressures—perhaps a channel with a higher mechanosensitive threshold or requiring higher levels of IP_3,_ DAG or Ca^2+^. Candidates include TRPV1, which has also been implicated in arterial myogenic constriction (Phan et al., 2022).

Although the deletion/inhibition of several TRP channel family members and other channels did not significantly impact pressure-induced chronotropy, it did impact other aspects of lymphatic contractile function, namely contraction amplitude and/or tone. The respective channels and their statistically significant contraction effects are summarized in Table 2. Deletion of TRPC6/TRPC3 impaired contraction amplitude by ~40% and increased tone by ~2.5-fold (Fig. 4C). Deletion of TRPV4 from LMCs impaired contraction amplitude by ~45% without an effect on tone (Fig. 7C). TRPV4 channel activation has recently been implicated in the regulation of lymphatic contraction, but primarily via the production of vasoactive products from LECs and resident macrophages (Schulz et al., 2025). There were three other channels whose inhibition led to significant changes in amplitude and/or tone. Specifically, one of the two TRPV2 inhibitors (SET2) inhibited tone at 4 of the 5 lowest pressures. One of the two ENaC inhibitors (amiloride) inhibited amplitude by 20–30% while the other one (benzamil) enhanced tone by ~2-fold (Fig. 9C). The Kir2 blocker Ba^2+^ impaired amplitude by ~40% and increased tone by 2-fold (Fig. 13C). Collectively, these effects suggest that the respective channels are each involved in some aspect of lymphatic contractile function (other than pressure-induced chronotropy), with the more compelling evidence being that obtained after by gene deletion rather than after acute application of soluble inhibitors, which may have off-target effects.

### Future directions.

Lastly, the critical mechanosensitive GNAQ/GNA11-coupled GPCR(s) in LMCs remain(s) to be identified. Although evidence points to AT_1_R as the key GNAQ/GNA11 -coupled mechanosensitive GPCR in arterial smooth muscle (Mederos y Schnitzler et al., 2011; Davis et al., 2023c), message for AT_1_R is negligible in LMCs (Fig. 18A) and neither *Agtr1* knock out or pharmacologic AT_1_R blockade altered pressure-induced chronotropy (Fig. 18B–C). No studies to date have attempted to identify the major GPCRs expressed in LMCs, e.g., by analyzing the various published scRNAseq databases for LMCs (Kenney et al., 2022; Lei et al., 2025; Schulz et al., 2025; Zawieja et al., 2025), but doing so might provide a starting point for identifying the specific mechanosensitive GNAQ/GNA11 - coupled (and other) GPCRs that mediate pressure-induced chronotropy.

### Physiological Relevance.

LMC function is impaired in Cantú syndrome, the only known example of primary lymphedema produced by a gene mutation (*Kcnj8/Abbc9*) in LMCs (Davis et al., 2023b). This impairment is evident both in lower contraction frequencies and amplitudes at multiple pressure levels. The lower frequency is consistent with parallel observations that gain-of-function mutations in K_ATP_ channels increase metabolic stress (Kim et al., 2024), whereas the lower amplitude suggests that other aspects of LMC function are altered as a consequence of chronic K_ATP_ channel hyperactivation. It is interesting that lymphatic contraction frequency and amplitude are also impaired in secondary lymphedema, in aging and in various models of metabolic disease [(Castorena-Gonzalez et al., 2025) and see discussion and references in (Davis et al., 2023e)]. Potential therapies for reversing LMC dysfunction under these conditions will depend on identifying the key ion channels and signaling pathways that drive pacemaking and control contraction amplitude. The present study represents a major step forward in the identification of specific LMC targets that could be used for that purpose.

## Supplementary Material

Supplement 1

## Figures and Tables

**Table 1.** Genes for which deletion/inhibition produced significant attenuation or enhancement of the F-P relationship.

Attenuation	Enhancement
*Cacna1c*	*TrpC6/TrpC3*
*Ano1*	-
*Itpr1*	-
*Gnaq*	-
*Gna11*	-
*Kcnj2/Kcnj12*	-

**Table 2.** Consequences of deletion/inhibition of ion channels on contraction amplitude and/or tone.*

Channel	Effect on AMP	Effect on Tone	Method of inhibition
TRPC6/3	↓	↓	deletion
TRPV4	↓	-	deletion
TRPV2	-	↓	soluble inhibitor
ENaC	↓	↑	soluble inhibitor
Kir2.x	↓	↑	soluble inhibitor

* Excluding the GNAQ/GNA11-IP_3_R1/Ca^2+^-ANO1-Cav1.2 signaling pathway.

## Data Availability

RNA sequence data were deposited in GEO (accession number GSE277843). All data generated or analyzed during this study are included in the manuscript and supporting files; source data files have been provided for all figures.
