# Supplementary material for: Roles of G-protein coupled receptors and mechanosensitive ion channels in pressure-induced chronotropy of lymphatic vessels
Source: bioRxiv. 2025 Oct 7:2025.10.07.681001. Preprint. [Version 1] doi: 10.1101/2025.10.07.681001 (PMC12632312; doi:10.1101/2025.10.07.681001)
Supplement: Supplement 1 [file NIHPP2025.10.07.681001v1-supplement-1.pdf]

bioRxiv preprint doi: https://doi.org/10.1101/2025.10.07.681001; this version posted October 7, 2025. The copyright holder for this preprint (which was not certified by peer review) is the author/funder, who has granted bioRxiv a license to display the preprint in perpetuity. It is made available under aCC-BY 4.0 International license.

Suppl. Table 1. Primers used for qPCR.

Gene	Accession Number	Catalog Number	Description
Ano1	NM_178642	IDT Mm.PT.58.12522115	Mouse Ano1 TaqMan probe
Acta2	NM_007392	IDT Mm.PT.58.16320644	Mouse α-actin TaqMan probe
β-Actin	NM_007393	IDT Mm.PT.58.33257376.gs	Mouse Actb TaqMan probe
